# A Serosurvey for Ruminant Pestivirus Exposure Conducted Using Sera From Stray Mexico Origin Cattle Captured Crossing Into Southern Texas

**DOI:** 10.3389/fvets.2022.821247

**Published:** 2022-03-15

**Authors:** Shollie M. Falkenberg, Fernando V. Bauermann, Glen A. Scoles, Denise Bonilla, Rohana P. Dassanayake

**Affiliations:** ^1^United States Department of Agriculture (USDA), Agricultural Research Service (ARS), Ruminant Disease and Immunology Research Unit, National Animal Disease Center, Ames, IA, United States; ^2^Department of Veterinary Pathobiology, College of Veterinary Medicine, Oklahoma State University, Stillwater, OK, United States; ^3^USDA, ARS, Animal Disease Research Unit, Washington State University, Pullman, WA, United States; ^4^USDA, Animal and Plant Health Inspection Service (APHIS), Veterinary Services, Fort Collins, CO, United States

**Keywords:** bovine viral diarrhea virus, cattle, HoBiPeV, virus neutralizing titer, pestivirus, seroprevalence

## Abstract

The US Department of Agriculture (USDA), Animal Plant Health Inspection Service (APHIS), Cattle Fever Tick Eradication Program (CFTEP) monitor a quarantine zone along the Texas border to prevent the introduction of stray livestock carrying cattle fever ticks entering the United States from Mexico. Stray cattle collected by CFTEP are checked for ticks and several infectious disease-causing pathogens, but not for bovine viral diarrhea virus (BVDV). BVDV is one of the most economically impactful viruses affecting US cattle producers. BVDV is present in all parts of the world, but it has been demonstrated that another distantly related pestivirus, HoBi-like pestivirus (HoBiPev), can also cause BVD. To date, HoBiPev has not been detected in the United States, but is commonly found in Brazil, and sporadically in Europe and Asia. The objective of the current study was to evaluate the seroprevalence of pestiviruses, with a specific focus on HoBiPev, in stray cattle. Virus neutralization (VN) assay was used to determine seroprevalence (or antibody titers) of BVDV-1, BVDV-2, and HoBiPev. Approximately 50% (67 of 134) of the samples were seropositive for pestiviruses; all 67 positive samples were positive (50%) for BVDV-1, 66 samples of the 67 were positive (49.3%) for BVDV-2, and the same 66 samples of the 67 were also positive (49.3%) for HoBiPev. Due to the antigenic cross-reactivity among Pestiviruses, the comparative antibody against each pestivirus was calculated from all VN-positive samples. Titers were clearly higher against BVDV-1, and only one sample had a titer clearly higher against BVDV-2. No sample had an antibody titer higher for HoBiPev, and while this does not prove the absence of HoBiPev, it does provide evidence that the prevalence of HoBiPev is less predominant than BVDV-1. Additionally, data from these samples provide evidence on the susceptibility of animals that may enter into the United States, with ~50% of the animals seronegative for bovine pestiviruses. This cattle population provides a unique opportunity to evaluate and monitor changes in seroprevalence of economically important cattle diseases affecting the cattle industry.

## Introduction

The US cattle industry was devastated in the beginning of the 20th century by bovine babesiosis, also known as cattle fever or Texas cattle fever. This tick-borne disease is caused by *Babesia bovis* and *Babesia bigemina* (1). A concerted effort to eradicate the tick vector was undertaken by establishment of quarantine lines and treating all cattle moving from south to north over these lines. Progressively, the quarantine line was moved farther south each year until cattle fever tick eradication was achieved in the United States. In 1943, the last population of cattle fever ticks were eradicated from Texas, and the current permanent quarantine line was established along the border with Mexico to prevent reinvasion (2). While more than 1 million cattle are legally imported into the United States from Mexico and are evaluated for a variety of pathogens, stray cattle along the TX/Mexico border that occasionally cross the border create an opportunity for the introduction of pathogens. The United States Department of Agriculture (USDA), Animal and Plant Health Inspection Service (APHIS), and Cattle Fever Tick Eradication Program (CFTEP) monitor the premises adjacent to the Mexico border in counties that comprise the permanent quarantine zone for detection and management of stray cattle (3). When observed, these cattle are apprehended and evaluated for a variety of pathogens similar to that of legally imported cattle. The numbers of stray cattle apprehended may be influenced by a variety of factors, such as violence along the border, environmental effects (drought, weather patterns, temperature, etc.), river levels, and economic or financial hardships (4). Monitoring this population of stray cattle provides a unique opportunity to evaluate the risk of introducing pathogens that potentially pose a threat to the US cattle industry.

The *Pestivirus* genus (family *Flaviviridae*) contains viruses of importance for the cattle industry, namely, bovine viral diarrhea virus (BVDV)-1, BVDV-2, and the more recently described HoBiPev. These viruses are classified as *Pestivirus A, B*, and *H* species, respectively (5). The most predominant subgenotype detected in BVDV persistently infected (PI) calves in the United States is BVDV-1b (6, 7), with BVDV-1a and 2a making up the other majority subgenotypes. Recently, other genetically diverse BVDV-1 and 2 isolates belonging to 1c, 1i, 2b, and 2c subgenotypes have also been identified in the United States (8, 9). The recent identification of BVDV-1c in the United States is interesting given that BVDV-1c is the most predominate BVDV subgenotype in Mexico and also one of the most predominate BVDV-1 subgenotype globally (10, 11). To date, HoBiPev has only been detected in South America, Europe, and Asia but not in North America (12–16).

The intent of a serological survey is not to identify active infections, but rather detecting previous exposure to a particular pathogen. While this does not give a present perspective on what is currently circulating, it does provide a general representation of the presence of a particular pathogen in the population. Therefore, the objective of the current study was to test sera collected from stray cattle along the Texas/Mexico border for neutralizing antibodies against reference BVDV-1a, BVDV-2a, and HoBiPev strains. Samples seropositive against the BVDV-1a reference virus were subsequently tested against a BVDV-1c virus for comparative titer evaluation as a method to better understand if the source of exposure was natural given that BVDV-1c was the most prevalent subgenotype in Mexico.

## Materials and Methods

### Animals and Sample Collection

All serum samples were initially shipped on ice to USDA, Agricultural Research Service (ARS), Animal Disease Research Unit (ADRU) in Pullman, WA, USA. Upon arrival, the samples were stored at −20°C, subsequently thawed to obtain an aliquot for pestivirus serology, and shipped frozen to USDA-ARS-National Animal Disease Center (NADC). Samples were maintained at −20°C until virus neutralization (VN) assays were completed. Paired blood samples from this group of cattle were also tested in Pullman for tick-borne pathogens; those results are reported separately (3).

Research activities involving animals conducted by the USDA were carried out in accordance with the guidelines set forth in the “Guide for the Care and Use of Agricultural and Use of Laboratory Animals” (8th Edition, 2011), and the “Guide for the Care and Use of Agricultural Animals in Research and Teaching” (4th Edition, 2020). All animal procedures were approved by each respective location-specific Institutional Animal Care and Use Committee(s) where the samples were collected. Detailed animal handling procedures were previously described (3).

Samples were collected by APHIS personnel working along the Texas/Mexico border between Del Rio and Pharr, TX, USA, from five different stations (3). Blood collection tubes without additives were used for sample collection using the Vacutainer system. Serum samples from 134 stray cattle were collected and shipped to Pullman over the course of 15 months from October 2017 through December 2018 and then shipped together as a batch to Ames where they were available for VN assays. The information available for each animal is limited and includes sex, approximate age, and location. The 134 samples were from all cattle apprehended over the period from October 2017 through December 2018, not just a subset of cattle. Additionally, the average number of cattle apprehended each year from 2010 to 2021 is ~80 head of cattle (Cattle Fever Tick Eradication Program; USDA/APHIS/Veterinary Services), making this a higher-than-average number of cattle apprehended and representative sample population.

### Virus

All viruses used for VN assays were propagated in Madin–Darby bovine kidney (MDBK) cells that had been tested and found free of BVDV and HoBiPev as previously described (14). Pestivirus isolates used included the cytopathic (cp) strains BVDV-1a Singer, BVDV-2a 296c, and HoBiPev Italy-1/101, and subsequently, BVDV-1c Bega was used for BVDV-1 comparative titer differentiation. Cell cultures in flasks, about 70% confluent, were inoculated with the pestivirus strains and frozen when over 85% of the cells were presenting a cytopathic effect (CPE). Cell culture flasks were freeze thawed, and culture medium was clarified by centrifugation at 500 × *g* for 10 min and passed through a 0.22-μm filter. Viral titers were determined via serial dilution on bovine turbinate (BTu) cells (17). Endpoint titers were determined based on observance of CPE and titers calculated based on the Spearman–Kärber method. MDBK cells used for virus propagation were grown in complete cell culture medium composed of minimal essential media (MEM; Sigma-Aldrich, St. Louis, MO, USA), whereas BTu cells were grown in DMEM. Cell culture media were supplemented with L-glutamine (1.4 mM; Gibco, Life Technologies, Grand Island, NY, USA), 1% of antibiotic–antimycotic-100X (Invitrogen, Life Technologies, Carlsbad, CA, USA), and 10% FBS (PAA, Ontario, Canada) that was heat inactivated. FBS was tested and found to be free of BVDV and HoBiPev, and antibodies against BVDV or HoBiPev.

### Virus Neutralization Assay

VN assays were performed as previously described (18) using the serum samples received from ADRU in Pullman, WA, USA. Prior to use in the VN assay, serum samples were thawed, filtered through a 0.2-μm membrane syringe filter, and placed in a water bath at 60°C for 60 min for inactivation of the complement system. Inactivated samples were maintained at 4°C until use in VN assays. Briefly, sera were tested by VN assay performing serial twofold dilutions of each antiserum in MEM, starting from a 1:8 initial dilution to 16,384 in 96-well microplates. Replicates of three wells for each serum dilution was used. A 50-μl aliquot of diluted serum and a 50-μl aliquot of virus containing 100 tissue culture infectious dose (TCID_50_) were added to each well and incubated for 1 h at 37°C. At the end of the incubation period, 20,000 BTu cells in a 100-μl aliquot of MEM and 10% FBS were added to each well. Microplates were placed in an incubator for 4 days at 37°C with a 5% CO_2_ concentration. Growth of the virus was evaluated by CPE, and plates were read using an optical microscope. Wells without any observed CPE in each serum dilution were used for the calculation of the endpoint titer through the Spearman–Kärber method, as previously described (19).

### Data Analysis

The animal information collected on apprehended cattle and VN test results were entered into an electronic spreadsheet. For the purpose of this study, the county information was entered as the country in which the stray cattle were processed and do not necessarily provide the exact location of apprehension. The neutralization results of the three wells at each respective dilution were evaluated for neutralization or lack of CPE. These results were averaged for the virus neutralization titer and were used to convert to a log_2_ value according to the Spearman–Karber method and used in determining the comparative ratio (R) for each serum sample. Neutralization titers that were undetectable at 1:8 dilution (<8) was considered to be negative.

Given the cross-reactivity of pestiviruses, the sera had titers against all three pestivirus species, but the antibody levels were not equal among the species. The better quantify the predominant titer among the three pestivirus species, the following formula, as previously reported (18), was used to determine the comparative ratio (R) for each sample. R_speciesX_ = (titer against species X + titer against species Y + titer against species Z)/(3 × titer against species X). Calculated ratios based on the previous formula were used to determine the predominant titer for each serum sample. A value higher than 0.2 when subtracted from the ratio value for the other two pestiviruses was used as the criteria to determine if the titer was substantially higher than the other titer(s). When the value was below 0.2 when subtracted from the ratio value for the other two pestiviruses, the sample was considered equivocal.

## Results

Samples with a titer <8 were considered to be negative for pestivirus antibodies. Fifty percent (*n* = 67) of the samples tested were seropositive against at least one of the pestiviruses. A small portion of samples had low level of antibodies (<16) against BVDV-2 (5.2%) and HoBiPev (1.5%) (Table 1). Conversely, titers >1,024 were only detected against the BVDV-1a strain (4.5%). Additionally, the percentage of samples that had titers ranging from 256 to <1,024 was highest against BVDV-1a (29.9%) and only 0.7 and 1.5% against BVDV-2 and HoBiPev, respectively (Table 1).

**Table 1:** Overall percentage of samples stratified by titer for *Bovine viral diarrhea virus 1* and *2* (BVDV-1 and−2) and HoBi-like virus.

	**Percent positive samples**		
**Titer group**	**BVDV-1a**	**BVDV-2a**	**HoBi-like**
<8	50.0	50.7	50.7
8 to <16	0.0	5.2	1.5
16 to <64	1.5	26.1	23.9
64 to <256	14.2	17.2	22.4
256 to <1,024	29.9	0.7	1.5
>1,024	4.5	0.0	0.0

Approximately 50.7% had no neutralizing titer for any tested pestivirus, whereas BVDV-1a titer predominated in 43.3% of the samples and BVDV-2 in 0.7% of the samples. Equivocal samples, with no predominate titer, were 5.2%, and no sample had a titer predominant for HoBiPev.

Titers detected against each of the pestivirus species varied by age (Table 2). In general, cattle that ranged in age from 5 to <10 years comprised the highest percentage of positive samples, whereas cattle <1 year of age had the highest proportion of samples that were negative for all three pestiviruses tested.

**Table 2:** Percentage of animals for different age groups (by year) stratified by titer against bovine viral diarrhea virus 1 and *2* (BVDV-1 and−2) and HoBi-like virus.

	**Age category (by year)**			
	** <1**	**1 to <5**	**5 to <10**	**>10**
**BVDV-1a**				
<8	6.7	8.2	26.9	8.2
8 to <16	0.0	0.0	0.0	0.0
16 to <64	0.7	0.0	0.7	0.0
64 to <256	0.0	2.2	11.9	0.0
256 to <1,024	0.0	4.5	24.6	0.7
>1,024	0.0	0.0	4.5	0.0
**BVDV-2a**				
<8	7.5	8.2	26.9	8.2
8 to <16	0.0	0.0	0.0	0.0
16 to <64	0.0	3.7	21.6	0.7
64 to <256	0.0	3.0	14.2	0.0
256 to <1,024	0.0	0.0	0.7	0.0
>1,024	0.0	0.0	0.0	0.0
**HoBi-like**				
<8	7.5	8.2	26.9	8.2
8 to <16	0.0	0.0	1.5	0.0
16 to <64	0.0	2.2	20.9	0.7
64 to <256	0.0	4.5	17.9	0.0
256 to <1,024	0.0	0.0	1.5	0.0
>1,024	0.0	0.0	0.0	0.0

**Figure 1:**
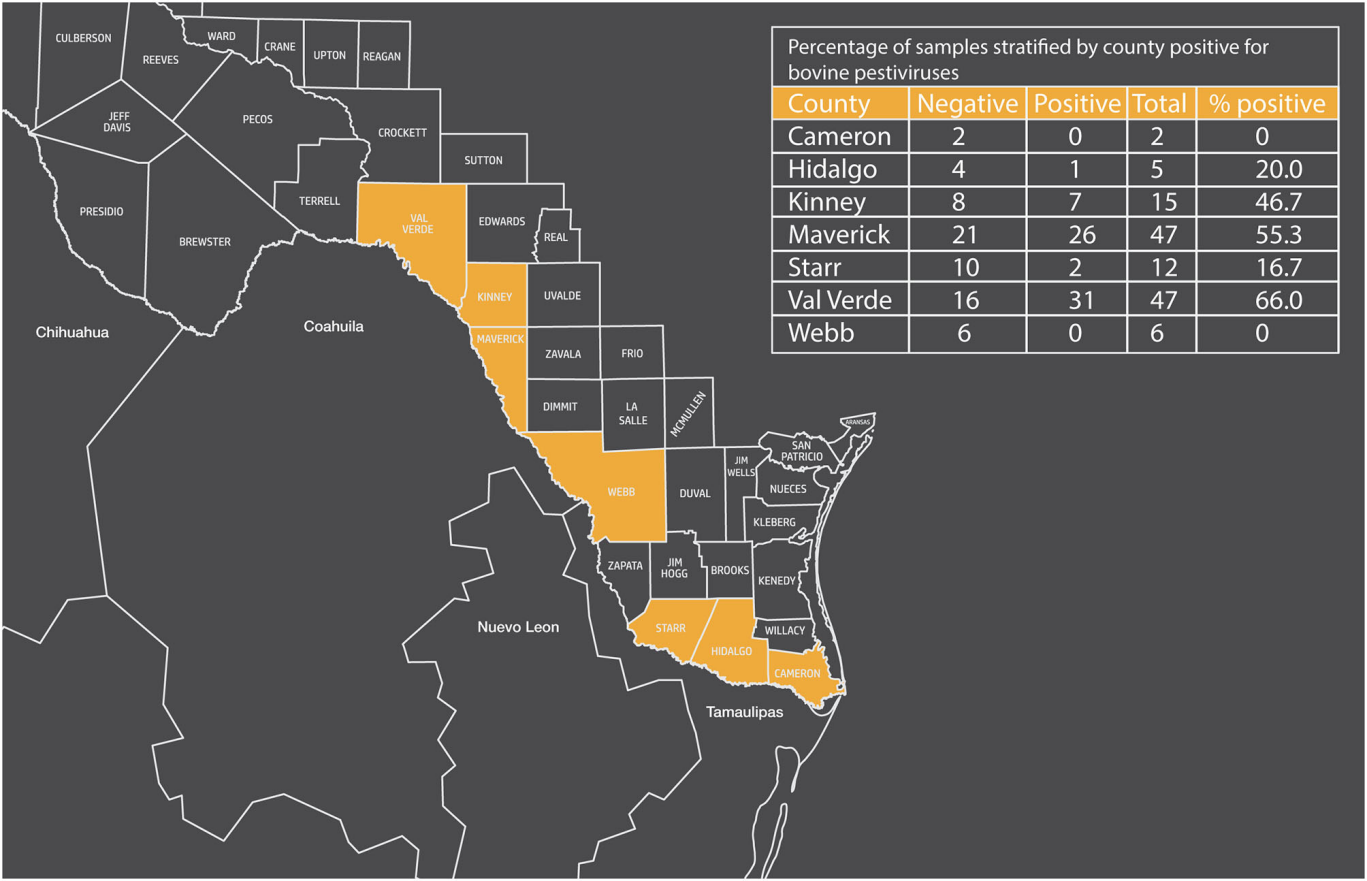
Map of Texas showing border counties and percentage of samples stratified by county positive for bovine pestiviruses.

Cattle seropositive against bovine pestiviruses did vary by location. Over 50% of the samples collected from Maverick and Val Verde counties were positive for bovine pestiviruses, and 46% of the samples from Kinney county were positive for bovine pestiviruses (Figure 1). In contrast, ≤ 20% of the samples were positive for bovine pestiviruses from the other four counties (Cameron, Hidalgo, Starr, and Webb).

Given that BVDV-1 titers were the predominant titer, all seropositive samples were subsequently re-evaluated for comparative titers between BVDV-1a and 1c. This was done to assess if the source of pestivirus exposure was by natural or vaccination since BVDV-1a viruses are in a vaccine and BVDV-1c is the predominate subgenotype in Mexico prevalence studies. Therefore, in a retrospective analysis, samples seropositive against BVDV-1a reference virus (*n* = 67) were tested against a BVDV-1c virus for comparative titer evaluation. The BVDV-1a titer predominated in ~28% of the samples, whereas ~72% of the samples were equivocal with no predominant titer. No sample had a titer predominant for BVDV-1c. While the 72% of the samples tested were equivocal, the BVDV-1a VNTs were higher than the BVDV-1c VNT.

## Discussion

Serologic results from stray Mexico cattle suggest that half of these cattle have been exposed to bovine pestiviruses. While the source of the exposure cannot be conclusively determined, the BVDV-1a titer predominated in the majority of samples that were seropositive. Given that BVDV-1c is the most predominant subgenotype circulating in Mexico, samples were subsequently evaluated against a BVDV-1c strain for comparative titers between BVDV-1a and BVDV-1c to better evaluate the source of the exposure. The majority of the comparative titer values for samples seropositive against the BVDV-1a reference virus were equivocal, and neither BVDV-1a nor BVDV-1c titer predominated. Although no sample had a titer predominant for BVDV-1c, in 28% of the samples tested, the BVDV-1a titer predominated. Additionally, the average BVDV-1a VNT were higher than the average BVDV-1c VNT. Collectively, these data would suggest that the exposure may be associated with vaccination rather than natural exposure to BVDV. Given that, currently, vaccination is considered good livestock practice, but no data are available to determine the actual vaccine usage (20), the route of pestivirus exposure cannot be conclusively determined, although the three Mexican states (Coahuila, Nuevo Leon, and Tamaulipas) that border the permanent quarantine zone make up over 30% of the Mexico cattle exports, which, in turn, can lead to an increased risk for managed or processed Mexican cattle escaping enclosures or breaking free from larger groups (4, 21). During drought periods, ranchers may move their animals closer to the river (Rio Grande) in search of forage, which may facilitate cattle unintentionally crossing the river while grazing. Additionally, small farm/backyard cattle are at greater risk of becoming lost or stray during droughts, financial hardship, periods of violence, or may lead to farm abandonment (4, 22).

No sample had an antibody titer profile that was substantially higher for HoBiPev; this does not prove the absence of the virus in the United States or Mexico. It does indicate that HoBiPev, if present, are much less predominant than BVDV-1 or BVDV-2. Serological surveys do not give a present perspective on what is currently circulating; it does provide information on the level of susceptibility of a group. The overall percentage of animals with titers below levels that are considered to be protective against acute bovine pestivirus infection (titer ≤ 16) was 50% for BVDV-1a, 55.9% for BVDV-2a, and 52.2% for HoBiPev (Table 1). Therefore, over half of these animals are susceptible when considering titers to protect against fetal infections (titer ≥256). The percentage of susceptible animals is 65.7% for BVDV-1a, 99.2% for BVDV-2a, and 98.5% for HoBiPev (Table 1). Comparing titers against different BVDV-1 strains, as well as BVDV-2 and HoBiPev, yield important information regarding the predictive impact of the introduction of emerging pestiviruses. Data from this study are concerning given that most stray cattle that have the ability to cross between the United States and Mexico are a potential susceptible population for pestivirus infections and could be a source of transmission of pestiviruses.

Seroprevalence studies in cattle have been performed both in the United States and Mexico, providing a general overview for each respective country and the risk of introducing a novel pestivirus due to unaccounted-for stray cattle that could move reciprocally across the country border. In a recent US seroprevalence study, a large portion of samples were reported to be seropositive for ruminant pestiviruses, 91.3% for BVDV-1, 89.3% for BVDV-2, and 84.9% for HoBiPev (18). The titers tended to be higher against BVDV-1 and BVDV-2 suggesting a lack of evidence that HoBiPev is circulating in the United States. The first study of BVD in Mexico was a serosurvey in 1975 based on 47 nonvaccinated animals with a clinical history of abortions, infertility, and respiratory signs and reported seropositivity of 75% (23). Subsequent seroprevalence studies of dairy cattle and beef cattle from multiple states reported seropositivity to range from ~47 to 80% (20, 24–28). Therefore, in general, the seroprevalence rate in stray cattle tends to be lower than other seroprevalence surveys conducted in the United States and Mexico and provides further evidence of a more susceptible population of cattle that may cross country borders.

The age of the cattle apprehended and evaluated in this study ranged from a few months to over 10 years of age. This is a wide range in ages leading to a greater potential for exposure, both natural and from vaccination, whereas the most recent serosurvey in the United States did not evaluate cattle under 2 years of age, and in general, a greater percent and higher titers were observed in that study (18). In the current study, cattle that ranged from 5 to <10 years of age accounted for the largest seropositive samples as well as highest titers, whereas cattle <1 year and >10 years of age had the highest proportion of seronegative samples. Additionally, samples collected from Maverick, Val Verde, and Kinney counties had the greatest number of pestivirus seropositive samples, but these counties also had the greatest number of cattle apprehended between 5 and <10 years of age. Interestingly, these samples also had the greatest number of blood samples that also tested positive for tick-borne pathogens (3), suggesting that there may be an age associated with pathogen exposure in stray cattle. Due to the potential for the greater probability of environmental, financial, or violence to occur over a 10-year period, older cattle may have a greater likelihood of exposure to pathogens over the course of their life and, therefore, may be an age-associated exposure.

There is no way the source of the antibodies or the source of exposure can conclusively be determined. Further compounding the understanding of the source of exposure is the wildlife–livestock interface and the ability of pestiviruses to infect even-toed ungulates, which represents an extensive group of wildlife species. The stray cattle along the Texas/Mexico border and other animals that could move freely and travel across country lines without oversight are particularly important. Due to the inability to monitor and regulate the movement of these animals, they have the potential to introduce novel or new pathogens into areas unknowingly. Livestock can be controlled to a greater extent with man-made barriers, but wildlife present a greater challenge in managing the wildlife–livestock interface. Previous research has demonstrated that free-ranging white-tailed deer are seropositive and have been exposed to BVDV (29–31). While there are anecdotal reports of close contact between white-tailed deer and cattle, a survey conducted by USDA reports >50% of producers reporting the potential for physical or environmental contact between wild cervids and cattle (30, 32). Additionally, movement patterns of nilgai antelope have demonstrated the risk of wild cervids in contributing to the potential spread of pathogens that have an ability to infect both cervids and cattle (33). Together, both current and previous data emphasize the need to monitor animals that are apprehended and have the ability to cross borders. Evaluation of these animals provide a unique opportunity to assess the risk and monitor the potential introduction of novel pathogens. Regular monitoring of exposure rates and potential changes in serological trends can provide insights into the introduction of emerging or novel pestiviruses.

## Data Availability Statement

The raw data supporting the conclusions of this article will be made available by the authors, without undue reservation.

## Ethics Statement

All animal procedures were approved by each respective location specific Institutional Animal Care and Use Committee(s) where the samples were collected.

## Author Contributions

SF and FB: conceived and designed the experiment. SF and RD: performed the experiment. SF, RD, and FB: analyzed the data. GS and DB: contributed reagents, materials, and analysis tools. SF: wrote the paper. FB, GS, DB, and RD: reviewed the paper. All authors contributed to the article and approved the submitted version.

## Funding

This research was conducted at a USDA research facility without external support.

## Conflict of Interest

The authors declare that the research was conducted in the absence of any commercial or financial relationships that could be construed as a potential conflict of interest.

## Publisher's Note

All claims expressed in this article are solely those of the authors and do not necessarily represent those of their affiliated organizations, or those of the publisher, the editors and the reviewers. Any product that may be evaluated in this article, or claim that may be made by its manufacturer, is not guaranteed or endorsed by the publisher.
